# Supplementary material for: The Systems Biology Research Tool: evolvable open-source software
Source: BMC Syst Biol. 2008 Jun 29;2:55. doi: 10.1186/1752-0509-2-55 (PMC2446383; doi:10.1186/1752-0509-2-55)
Supplement: Additional file 1 — SBRT Archive. An archive of the current version of the Systems Biology Research Tool. [file 1752-0509-2-55-S1.zip › sbrt-1.4.0/doc/developers_guide/api/sbrt/kernel/math/AbstractLinearComb.Term.html]

AbstractLinearComb.Term


|  |  |  |
| --- | --- | --- |
| **Overview** \| **Package** \| **Class** \| **Use** \| **Tree** \| **Deprecated** \| **Index** \| **Help** | |  |
| **PREV CLASS**   **NEXT CLASS** | **FRAMES**    **NO FRAMES**     **All Classes** |  |
| SUMMARY: NESTED \| FIELD \| CONSTR \| METHOD | DETAIL: FIELD \| CONSTR \| METHOD |  |


---


## sbrt.kernel.math Class AbstractLinearComb.Term<V>

```
java.lang.Object
  sbrt.kernel.math.AbstractLinearComb.Term<V>
```

**Type Parameters:**: `V` - the variable type.

**All Implemented Interfaces:**: java.lang.Iterable<V>, LinearComb.Term<V>, Polynomial.Term<V>

**Direct Known Subclasses:**: HashLinearComb.Term

**Enclosing class:**: AbstractLinearComb<V>

---

``` public abstract static class AbstractLinearComb.Term<V> extends java.lang.Object implements LinearComb.Term<V> ```

This class is a skeleton implementation of `LinearComb.Term`.

**Author:**
:   This class was written and documented by Jeremiah Wright while in
    the Wagner lab.

---

**Constructor Summary**

`AbstractLinearComb.Term(double coefficient,
V variable)`
  
          Constructs a new linear combination term with the provided
coefficient and variable.

`AbstractLinearComb.Term(V variable)`
  
          Constructs a new linear combination term with the provided
variable.

`AbstractLinearComb.Term(V variable,
double coefficient)`
  
          Constructs a new linear combination term with the provided
coefficient and variable.


| **Method Summary** | |
| --- | --- |
| `boolean` | `equals(java.lang.Object obj)`             Indicates if the provided object is equal to this term. |
| `double` | `getCoefficient()`             Returns the coefficient of this term. |
| `V` | `getVariable()`             Returns the variable contained in this term. |
| `java.util.Set<V>` | `getVariables()`             Returns the set of variables contained in this term. |
| `boolean` | `hasAllVariables(java.util.Collection<?> variables)`             Indicates if this term contains all of the variables in the provided collection. |
| `int` | `hashCode()`             Returns a content-based hash code for this term. |
| `boolean` | `hasVariable(java.lang.Object variable)`             Indicates if the provided object is a variable contained in this term. |
| `boolean` | `hasVariables()`             Returns `true`. |
| `java.util.Iterator<V>` | `iterator()`             Returns an iterator over the variables in this term. |
| `int` | `power(java.lang.Object variable)`             Returns the power of the provided variable in this term. |
| `java.lang.String` | `toString()`             Returns an appropriate string representation of this term. |
| `int` | `variables()`             Returns `1`. |

**Methods inherited from class java.lang.Object**

`clone, finalize, getClass, notify, notifyAll, wait, wait, wait`

**Constructor Detail**

### AbstractLinearComb.Term

```
public AbstractLinearComb.Term(double coefficient,
                               V variable)
```

:   Constructs a new linear combination term with the provided
    coefficient and variable.

    **Parameters:**: `coefficient` - the coefficient of this term.: `variable` - the variable of this term.

---


### AbstractLinearComb.Term

```
public AbstractLinearComb.Term(V variable,
                               double coefficient)
```

:   Constructs a new linear combination term with the provided
    coefficient and variable.

    **Parameters:**: `variable` - the variable of this term.: `coefficient` - the coefficient of this term.

---


### AbstractLinearComb.Term

```
public AbstractLinearComb.Term(V variable)
```

:   Constructs a new linear combination term with the provided
    variable. The coefficient equals 1.

    **Parameters:**: `variable` - the variable of this term.


**Method Detail**

### getVariable

```
public V getVariable()
```

:   Returns the variable contained in this term.

    :   **Specified by:**: `getVariable` in interface `LinearComb.Term<V>`
    :   **Returns:**: the variable contained in this term.

---


### getCoefficient

```
public double getCoefficient()
```

:   Returns the coefficient of this term.

    :   **Specified by:**: `getCoefficient` in interface `Polynomial.Term<V>`
    :   **Returns:**: the coefficient of this term.

---


### equals

```
public boolean equals(java.lang.Object obj)
```

:   Indicates if the provided object is equal to this
    term.

    Two terms are considered equal if they contain
    the same coefficient and variable.

    :   **Specified by:**: `equals` in interface `Polynomial.Term<V>` **Overrides:**: `equals` in class `java.lang.Object`
    :   **Parameters:**: `obj` - the object to be tested for equality with this term. **Returns:**: `true` if this term is equal to the provided object; `false` otherwise.

---


### hashCode

```
public int hashCode()
```

:   Returns a content-based hash code for this term.

    :   **Specified by:**: `hashCode` in interface `Polynomial.Term<V>` **Overrides:**: `hashCode` in class `java.lang.Object`
    :   **Returns:**: a content-based hash code for this term.

---


### toString

```
public java.lang.String toString()
```

:   Returns an appropriate string representation of this
    term.

    :   **Specified by:**: `toString` in interface `Polynomial.Term<V>` **Overrides:**: `toString` in class `java.lang.Object`
    :   **Returns:**: an appropriate string representation of this term.

---


### iterator

```
public java.util.Iterator<V> iterator()
```

:   Returns an iterator over the variables in this term.

    :   **Specified by:**: `iterator` in interface `java.lang.Iterable<V>` **Specified by:**: `iterator` in interface `Polynomial.Term<V>`
    :   **Returns:**: an iterator over the variables in this term.

---


### power

```
public int power(java.lang.Object variable)
```

:   Returns the power of the provided variable in this
    term.

    :   **Specified by:**: `power` in interface `Polynomial.Term<V>`
    :   **Parameters:**: `variable` - the variable whose power is to be returned. **Returns:**: the power of the provided variable in this term. If the provided object is not a variable in this term, 0 is returned.

---


### getVariables

```
public java.util.Set<V> getVariables()
```

:   Returns the set of variables contained in this term.

    :   **Specified by:**: `getVariables` in interface `Polynomial.Term<V>`
    :   **Returns:**: the set of variables contained in this term.

---


### hasAllVariables

```
public boolean hasAllVariables(java.util.Collection<?> variables)
```

:   Indicates if this term contains all
    of the variables in the provided collection.

    :   **Specified by:**: `hasAllVariables` in interface `Polynomial.Term<V>`
    :   **Parameters:**: `variables` - the collection of variables whose presence in this term is to be tested. **Returns:**: `true` if each element of the provided collection is a variable in this term; `false` otherwise.

---


### hasVariable

```
public boolean hasVariable(java.lang.Object variable)
```

:   Indicates if the provided object is a variable contained
    in this term.

    :   **Specified by:**: `hasVariable` in interface `Polynomial.Term<V>`
    :   **Parameters:**: `variable` - the variable whose presence in this term is to be tested. **Returns:**: `true` if this term contains the provided variable; `false` otherwise.

---


### hasVariables

```
public boolean hasVariables()
```

:   Returns `true`.

    :   **Returns:**: `true`.

---


### variables

```
public int variables()
```

:   Returns `1`.

    :   **Specified by:**: `variables` in interface `Polynomial.Term<V>`
    :   **Returns:**: `1`.


---


|  |  |  |
| --- | --- | --- |
| **Overview** \| **Package** \| **Class** \| **Use** \| **Tree** \| **Deprecated** \| **Index** \| **Help** | |  |
| **PREV CLASS**   **NEXT CLASS** | **FRAMES**    **NO FRAMES**     **All Classes** |  |
| SUMMARY: NESTED \| FIELD \| CONSTR \| METHOD | DETAIL: FIELD \| CONSTR \| METHOD |  |


---
